# Multi-UAV-Assisted ISAC System: Joint User Association, Trajectory Design, and Resource Allocation

**DOI:** 10.3390/e27090967

**Published:** 2025-09-17

**Authors:** Jinwei Wang, Renhui Xu, Laixian Peng, Xianglin Wei

**Affiliations:** 1 The College of Communications Engineering, Army Engineering University of PLA, Nanjing 210007, China; wjwei0204@163.com (J.W.); lxpeng@hotmail.com (L.P.); 2 The 63rd Research Institute, National University of Defense Technology, Nanjing 210007, China; weixianglin@nudt.edu.cn

**Keywords:** unmanned aerial vehicle, integrated sensing and communication system, malicious jamming, alternating optimization, long short-term memory, user mobility

## Abstract

Unmanned aerial vehicle (UAV)-assisted integrated sensing and communication (ISAC) systems have developed rapidly in the sixth generation (6G) era. However, factors such as the mobility of ground users and malicious jamming pose significant challenges to systems’ performance and reliability. Against this backdrop, this paper designs a multi-UAV-assisted ISAC system model under malicious jamming environments. Under the constraint of sensing accuracy, the total communication rate of the system is maximized through joint optimization of user association, UAV trajectory, and transmit power. The problem is then decomposed into three subproblems, which are solved using the improved auction algorithm (IAA), dream optimization algorithm (DOA), and rapidly-exploring random trees-based optimizer algorithm (RRTOA). The global optimal solution is approached through the alternating optimization-based predictive scheduling algorithm (AOPSA). Meanwhile, this paper also introduces a long short-term memory (LSTM) network to predict users’ dynamic positions, addressing the impact of user mobility and enhancing the system’s real-time performance. Simulation results show that compared with the baseline scheme, the proposed algorithm achieves a 188% improvement in communication rate, which verifies its effectiveness and superiority.

## 1. Introduction

With the deep integration of wireless communication technology and intelligent sensing, integrated sensing and communication (ISAC) has become one of the core technologies for sixth generation (6G) communication systems [1,2]. By sharing resources such as spectrum and hardware, ISAC can achieve collaborative optimization of communication and sensing, significantly improving spectrum efficiency and system performance [3]. In recent years, unmanned aerial vehicles (UAVs) have demonstrated extensive potential in ISAC systems due to their flexible deployment, wide coverage, and line-of-sight (LoS) communication links [4]. Integrating ISAC modules onto UAVs can not only enhance resource utilization but also provide communication and sensing services for ground users simultaneously [5,6].

In a UAV-assisted ISAC system, UAV trajectory design, user scheduling, and resource allocation are crucial factors affecting system performance. Currently, numerous studies focus on utilizing a single UAV to provide ISAC services for users [7,8,9,10,11,12]. For instance, Liu et al. [7] proposed a joint optimization problem of user scheduling, transmit power, and UAV trajectory, aiming to maximize energy efficiency and minimize radar mutual information (MI) under the premise of satisfying user sensing fairness. Deng et al. [8] studied the UAV-assisted integrated periodic sensing and communication (IPSAC) mechanism. Under the premise of satisfying sensing frequency and beam pattern gain requirement, the authors jointly optimized UAV trajectory, user association, target sensing selection, and transmit beamforming to maximize system communication rate. Zhou et al. [9] proposed a joint optimization problem of UAV CPU frequency, UAV radar sensing power, user transmit power, and UAV trajectory, aiming to minimize the total energy consumption of the UAV and users. Liu et al. [10] jointly optimized UAV trajectory, task scheduling, and power allocation. Under the constraint of communication rate, they maximized the radar estimation rate. Zhu et al. [11] proposed a reconfigurable intelligent surface (RIS)-assisted ISAC system operating in the millimeter-wave band, which configures the radio propagation environment by altering the phase of radio signals to enhance communication transmission rates. Zhu et al. [12] investigated a simultaneous transmitting and reflecting reconfigurable intelligent surface (STAR-RIS)-assisted ISAC dual secure communication system. By jointly designing the base station’s transmit beamforming and receive filter, along with the STAR-RIS’s transmission and reflection coefficients, the system maximizes the long-term average secrecy rate of users while ensuring the echo signal-to-noise ratio threshold and user rate constraints.

However, constrained by size and power, a single UAV has limited service capability and coverage range, making it difficult to handle scenarios where the geographical locations of communication users or targets are widely distributed. This has driven in-depth research on multi-UAV-assisted ISAC systems [13,14,15,16]. For example, Zhang et al. [13] investigated the resource allocation problem in multi-UAV-assisted ISAC systems. By jointly optimizing the UAV trajectory, user association, and beamforming design, the sum of weighted bit rates of all ground users was maximized while ensuring the sensing signal gain of targets. Qin et al. [14] proposed a joint optimization problem of user association, UAV trajectory planning, and power allocation, aiming to maximize the minimum weighted spectral efficiency among UAVs. Then, the authors introduced centralized and distributed schemes for solving the optimization problem. Wang et al. [15] considered a scenario where multiple UAVs collaboratively detect targets and transmit the collected data to a central UAV. Regarding the communication and sensing scheduling problem, the authors formulated it into two optimization problems, with the objectives of maximizing the average utility function (MAUF) and minimizing the completion time (MCT), respectively. Liu et al. [16] introduced radar MI to measure the sensing performance of ISAC from the perspective of information entropy. To achieve fair communication, the authors maximized the minimum communication rate of each Internet of Things (IoT) node through joint optimization of node scheduling, transmit power, and the three-dimensional (3D) trajectory of UAVs.

Although the aforementioned studies have made significant progress in UAV-assisted ISAC systems, most of them assume that users are in a stationary state and do not fully consider the impact of user mobility on system performance. In fact, some work has begun to focus on ISAC systems in dynamic scenarios. For example, Al-habob et al. [17] investigated a downlink multi-user ISAC system with multiple mobile eavesdropping UAVs, aiming to maximize user secrecy rates and ensure sensing quality by designing predictive beamforming schemes. However, this work did not consider user mobility and employed only a single base station to provide ISAC services. Pan et al. [18] explored a multi-UAV cooperative ISAC dynamic system, which maximizes the positioning accuracy of moving targets while ensuring communication service quality by jointly optimizing trajectories, base station association, spectrum, and power resources. Nevertheless, these studies still do not address the realistic threat of malicious jamming.

As an active attacker, malicious jamming aims to weaken and disrupt the normal communication activities of communication parties through various means (e.g., signal interference, electromagnetic attacks, etc.), thereby degrading communication performance. Jammers use advanced electronic jamming equipment to emit noise signals that are similar to or within the same frequency band as the communication signals, severely reducing the signal-to-noise ratio at the receiver and leading to degraded or even interrupted communication quality. In ISAC systems, malicious jamming not only disrupts communication links but also indirectly affects sensing accuracy and exacerbates power allocation conflicts.

Currently, limited work has focused on UAV-assisted ISAC systems in malicious jamming environments. Liu et al. [19] studied a UAV-enabled ISAC system under malicious jamming attacks, which maximizes the minimum transmission rate of users by optimizing transmit beamforming while meeting sensing constraints. However, this work only considered a simple scenario with a single fixed UAV deployment, lacking an analysis of multi-UAV dynamic trajectory optimization and user mobility. Mei et al. [20] explored the minimization of age of information (AoI) in scenarios with malicious jamming UAVs, reducing AoI by jointly designing the trajectories and power of legitimate UAVs, but similarly without considering multi-UAV cooperation and user mobility. Therefore, under multiple constraints such as mobility, sensing requirements, communication guarantees, and malicious jamming, achieving efficient, robust, and dynamic trajectory planning and resource allocation for UAVs has become a critical problem that urgently needs to be addressed.

Unlike existing studies, the innovation of this paper lies in being the first to thoroughly investigate the joint optimization of user association, UAV trajectory design, and power allocation in multi-UAV-assisted ISAC systems under the complex scenario of coexisting dynamic users and malicious jamming attacks. This paper designs a multi-UAV-assisted ISAC system in a malicious jamming environment, where multiple UAVs provide downlink communication services while sensing environmental information around ground users and dynamically optimizing their trajectories to mitigate the impact of malicious jamming. Additionally, we introduce a long short-term memory (LSTM) network to predict user positions, thereby addressing the challenges posed by mobility. The main contributions of this paper are summarized as follows:

- A joint optimization problem is built to jointly optimize user association, UAV trajectory, and transmit power, aiming to maximize the total communication rate of all users under the constraint of sensing accuracy. Due to the non-convexity of the problem, an effective alternating optimization-based predictive scheduling algorithm (AOPSA) is put forward to solve it.
- We decompose the optimization problem into three subproblems, which are solved using the improved auction algorithm (IAA), dream optimization algorithm (DOA), and rapidly-exploring random trees-based optimizer algorithm (RRTOA).
- An LSTM network is introduced to capture the temporal characteristics of user positions, and it is combined with AOPSA to enhance the system’s real-time performance and optimization efficiency.
- A series of simulations are conducted to evaluate the performance of AOPSA. Simulation results demonstrate that LSTM can effectively predict user positions. Moreover, AOPSA outperforms four benchmarks with diverse parameter settings.

The remainder of this paper is organized as follows. Section 2 describes the multi-UAV-assisted ISAC system model under malicious jamming environments. In Section 3, we propose the joint optimization problem of user association, UAV trajectory, and UAV transmit power. Section 4 provides a detailed introduction to the proposed optimization algorithms. Section 5 presents the simulation results of the proposed algorithms and discusses these results. Finally, Section 6 concludes the paper.

## 2. System Model

**Figure 1:**
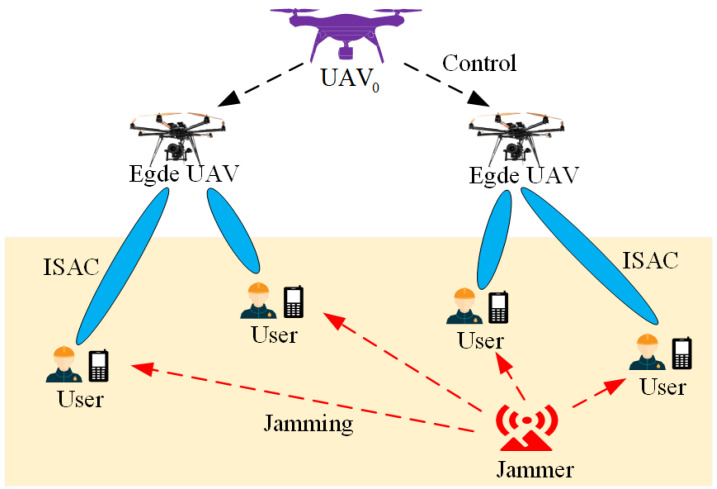
Multi-UAV-assisted ISAC system model under malicious jamming environments.

We consider a multi-UAV-assisted ISAC system under malicious jamming environments as shown in Figure 1. The system model includes the following: a central UAV, denoted as ${\mathrm{UAV}}_{0}$; *M* edge UAVs equipped with communication and sensing modules, denoted as ${\mathrm{UAV}}_{m}\in M=\left\{1,2,\ldots ,M\right\}$; *K* ground mobile users, denoted as ${\mathrm{UE}}_{k}\in K=\left\{1,2,\ldots ,K\right\}$; and a static malicious jammer. In this system, ${\mathrm{UAV}}_{0}$ provides global management and control, edge UAVs perform ISAC service for the users by sensing the environmental information around the users and sending the sensing information back to the users [16]. The jammer operates in a fixed-frequency mode, continuously emitting jamming signals within the primary communication frequency band (e.g., the ISAC shared band) to disrupt the communication link between edge UAVs and users. Such jammers are typically stationary (e.g., ground-based fixed jamming stations) and transmit at a constant power, generating strong interference via high-gain antennas. Due to the distance-dependent attenuation characteristics of wireless signal propagation, the jamming intensity experienced by user nodes is negatively correlated with their distance from the jammer. Based on feedback regarding the jamming intensity received by ground users, the UAV can perform radar detection scans over suspected jammer locations to estimate its approximate position [21,22].

Assume ${\mathrm{UAV}}_{0}$ hovers directly above the center of the area at a fixed altitude of $H_{0}$. Edge UAVs fly at a fixed altitude *H*, with flying time $T_{\mathrm{total}}$ divided into *T* time slots of length $\delta_{t}=\left(T_{\mathrm{total}}/T\right)$. $\delta_{t}$ should be sufficiently small to ensure sensing and communication channel state information (CSI) remain nearly constant. We consider a 3D Cartesian coordinate system. At each time slot t∈T={1,2,…,T}, the coordinates of ${\mathrm{UAV}}_{m}$ can be expressed as $q_{m}\left(t\right)={\left[x_{m}\left(t\right),y_{m}\left(t\right),H\right]}^{T}$, the coordinates of ${\mathrm{UE}}_{k}$ can be expressed as $q_{k}\left(t\right)={\left[x_{k}\left(t\right),y_{k}\left(t\right),0\right]}^{T}$, and the jammer’s coordinates can be expressed as $q_{J}={\left[x_{J},y_{J},0\right]}^{T}$. The motion constraints of ${\mathrm{UAV}}_{m}$ can be expressed as

(1) $$\begin{array}{c} ∥q_{m}\left(t+1\right)-q_{m}\left(t\right)∥\leq v_{\max}\delta_{t},\forall t \end{array}$$

(2) $$\begin{array}{c} ∥q_{m}\left(t\right)-q_{n}\left(t\right)∥\geq D_{\min},\forall t,m\neq n \end{array}$$

where ∥·∥ denotes the $L_{2}$ norm, $v_{\max}$ is the maximum flying speed of edge UAVs, and $D_{\min}$ represents the minimum collision avoidance distance between edge UAVs.

**Figure 2:**
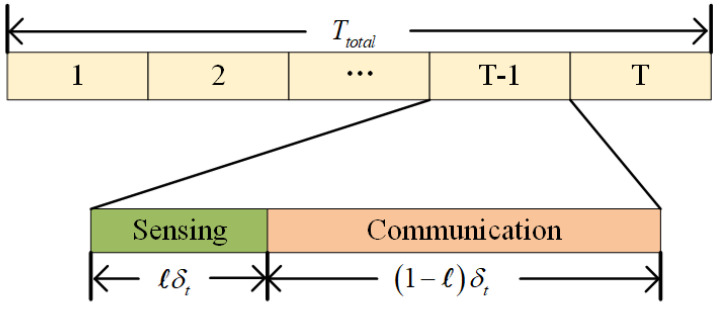
Time slot division.

To prevent mutual interference between radar signals and communication signals, the UAVs employ the time-division multiplexing (TDM) technique for radar detection and communication [16]. As depicted in Figure 2, each time slot is divided into two subtime slots by the allocation weight parameter *ℓ*. One subtime slot is allocated for the sensing task, while the other subtime slot is designated for the communication task.

We consider the air-to-ground communication channel mainly experiences Nakagami-m fading. Therefore, the communication channel gain from ${\mathrm{UAV}}_{m}$ to ${\mathrm{UE}}_{k}$ at time slot *t* can be expressed as [16,23]

(3) $$h_{mk}^{\mathrm{com}}\left(t\right)=\partial_{mk}\left(t\right)\frac{G_{s}G_{r}\lambda^{2}}{{\left(4\pi \right)}^{2}d_{mk}^{2}\left(t\right)}=\frac{\partial_{mk}\left(t\right)\beta_{\mathrm{com}}}{d_{mk}^{2}\left(t\right)}$$

where $\partial_{mk}\left(t\right)$ represents the small-scale fading coefficient following the Nakagami-m distribution, $G_{s}$ denotes the antenna gain of the transmitter of ${\mathrm{UAV}}_{m}$, $G_{r}$ denotes the antenna gain of the receiver of ${\mathrm{UE}}_{k}$, $d_{mk}\left(t\right)=∥q_{m}\left(t\right)-q_{k}\left(t\right)∥$ represents the distance between ${\mathrm{UAV}}_{m}$ and ${\mathrm{UE}}_{k}$, λ is the signal wavelength, and $\beta_{\mathrm{com}}=\left(G_{s}G_{r}\lambda^{2}\right)/{\left(4\pi \right)}^{2}$. Considering the transmit and return links of the radar signal, the channel power gain of the radar detection link ${\mathrm{UAV}}_{m}$ and ${\mathrm{UE}}_{k}$ can be expressed as

(4) $$h_{mk}^{\mathrm{rad}}\left(t\right)=\frac{G_{s}G_{e}\lambda^{2}\sigma }{{\left(4\pi \right)}^{3}d_{mk}^{4}\left(t\right)}=\frac{\beta_{\mathrm{rad}}}{d_{mk}^{4}\left(t\right)}$$

where $G_{e}$ denotes the antenna gain of the radar receiver of ${\mathrm{UAV}}_{m}$, σ represents the radar cross section (RCS) of the target, and $\beta_{\mathrm{rad}}=\left(G_{s}G_{e}\lambda^{2}\sigma \right)/{\left(4\pi \right)}^{3}$. Considering that the ground-to-ground communication channel mainly experiences Rayleigh fading, the jamming channel gain from the malicious jammer to ${\mathrm{UE}}_{k}$ can be expressed as [24]

(5) $$h_{Jk}^{\mathrm{jam}}\left(t\right)=\frac{G_{J}G_{r}\lambda^{2}}{{\left(4\pi \right)}^{2}d_{Jk}^{2}\left(t\right)}\psi =\frac{\beta_{\mathrm{jam}}}{d_{Jk}^{2}\left(t\right)}\psi$$

where $G_{J}$ denotes the antenna gain of the transmitter of the jammer, $d_{Jk}\left(t\right)=∥q_{J}-q_{k}\left(t\right)∥$ represents the distance between the jammer and ${\mathrm{UE}}_{k}$, ψ is a random variable following the exponential distribution with unit mean, and $\beta_{\mathrm{jam}}=\left(G_{J}G_{r}\lambda^{2}\right)/{\left(4\pi \right)}^{2}$.

In this work, the UAVs communicate with users via orthogonal frequency division multiple access (OFDMA). At time slot *t*, the signal-to-interference-plus-noise ratio (SINR) of the communication link between ${\mathrm{UAV}}_{m}$ and ${\mathrm{UE}}_{k}$ can be expressed as

(6) $$\gamma_{mk}^{\mathrm{com}}\left(t\right)=\frac{p_{m}\left(t\right)h_{mk}^{\mathrm{com}}\left(t\right)}{\sum_{l=1,l\neq m}^{M}p_{l}\left(t\right)h_{lk}^{\mathrm{com}}\left(t\right)+\sigma_{\mathrm{noise}}^{2}+p_{J}h_{Jk}^{\mathrm{jam}}\left(t\right)}$$

where $p_{m}\left(t\right)$ denotes the transmit power of ${\mathrm{UAV}}_{m}$ and satisfies $0\leq p_{m}\left(t\right)\leq p_{\max}$, and $p_{\max}$ is the maximum transmit power of edge UAVs. $\sigma_{\mathrm{noise}}^{2}$ is the additive white Gaussian noise (AWGN) power, and $p_{J}$ represents the transmit power of the jammer. The SINR of the sensing link between ${\mathrm{UAV}}_{m}$ and ${\mathrm{UE}}_{k}$ can be expressed as

(7) $$\gamma_{mk}^{\mathrm{rad}}\left(t\right)=\frac{p_{m}\left(t\right)h_{mk}^{\mathrm{rad}}\left(t\right)}{\sum_{l=1,l\neq m}^{M}p_{l}\left(t\right)h_{lk}^{\mathrm{rad}}\left(t\right)+\sigma_{\mathrm{noise}}^{2}}$$

To ensure the sensing accuracy of edge UAVs, the SINR of the sensing link needs to satisfy the constraint $\gamma_{mk}^{\mathrm{rad}}\left(t\right)\geq \gamma_{th}^{\mathrm{rad}}$ [25], where $\gamma_{th}^{\mathrm{rad}}$ represents the minimum SINR for accurate radar sensing.

We introduce a binary integer variable $\alpha_{mk}\left(t\right)$ to represent user association. If $\alpha_{mk}\left(t\right)=1$, it indicates that ${\mathrm{UAV}}_{m}$ and ${\mathrm{UE}}_{k}$ are associated at time slot *t*, otherwise $\alpha_{mk}\left(t\right)=0$. Assuming that each user can associate with at most one UAV at each time slot, it can be expressed as

(8) $$\alpha_{mk}\left(t\right)=\left\{\begin{array}{cc} 1, & \mathrm{if}\;{\mathrm{UAV}}_{m}\;\mathrm{and}\;{\mathrm{UE}}_{k}\;\mathrm{are}\;\mathrm{associated}\\ 0, & \mathrm{otherwise} \end{array}\right.$$

(9) $$\sum_{m=1}^{M}\alpha_{mk}\left(t\right)\leq 1,\;\forall k,t$$

At time slot *t*, the communication rate from ${\mathrm{UAV}}_{m}$ to ${\mathrm{UE}}_{k}$ can be expressed as

(10) $$R_{mk}^{\mathrm{com}}\left(t\right)=\log_{2}\left(1+\gamma_{mk}^{\mathrm{com}}\left(t\right)\right)$$

Therefore, the total communication rate of ${\mathrm{UE}}_{k}$ can be expressed as

(11) $$R_{k}^{\mathrm{com}}=\sum_{t=1}^{T}\sum_{m=1}^{M}\alpha_{mk}\left(t\right)\left(1-l\right)R_{mk}^{\mathrm{com}}\left(t\right)$$

The total communication rate of all users can be expressed as

(12) $$R_{\mathrm{sum}}^{\mathrm{com}}=\sum_{k=1}^{K}R_{k}^{\mathrm{com}}$$

## 3. Problem Formulation

Our goal is to maximize the total communication rate of all users through the joint optimization of user association, UAV trajectory, and UAV transmit power. The variables of user association, UAV trajectory, and transmit power are defined as follows: $\alpha =\{\alpha_{mk}\left(t\right),\forall m,k,t\}$, $q=\{q_{m}\left(t\right),\forall m,t\}$, and $p=\{p_{m}\left(t\right),\forall m,t\}$. The joint optimization problem can be formulated as follows

(13)P0:maxα,q,pRsumcom(13a)s.t.∑m=1Mαmk(t)≤1,∀k,t(13b)αmk(t)∈{0,1},∀m,k,t(13c)γmkrad(t)≥γthrad,∀m,k,t(13d)0≤pm(t)≤pmax,∀m,t(13e)xmin≤xm(t)≤xmax,∀m,t(13f)ymin≤ym(t)≤ymax,∀m,t(13g)∥qm(t+1)−qm(t)∥≤vmaxδt,∀t(13h)∥qm(t)−qn(t)∥≥Dmin,∀t,m≠n(13i)pm(t)·δt+κ·∥qm(t+1)−qm(t)∥≤Emax/T,∀m∈M,t∈T

where (13a) and (13b) are user association constraints, (13c) is the sensing accuracy constraint, (13d) is the UAV transmit power constraint, (13e) and (13f) are the UAV horizontal position constraints, (13g) and (13h) are the UAV motion trajectory constraints, and (13i) is the UAV energy consumption constraint, where κ is the flight energy consumption coefficient per unit distance and $E_{\max}$ is the maximum battery capacity of the UAV.

## 4. Algorithm Design

It is evident that problem P0 is a typical non-convex mixed integer nonlinear programming (MINLP) problem, which is challenging to solve directly. On one hand, the user association optimization variable α is a binary integer. On the other hand, even with fixed user association, the problem remains non-convex with respect to UAV trajectory q and transmit power p. Therefore, to solve problem P0, we decompose the original optimization problem into three subproblems: user association optimization, UAV trajectory optimization, and UAV transmit power optimization. Then, we solve these three subproblems through alternating iterations to obtain the solution of the original problem. Before introducing the algorithms, we first present the specific mechanism of LSTM for predicting user positions.

### 4.1. LSTM Predicting User Positions

Assuming ground users follow a random walk model and their current position information can be obtained through a global positioning system. The position information of *K* users over the past *S* time slots can be represented as

(14) $$I_{\mathrm{history}}=\left[\begin{array}{cccc} q_{1}\left(t_{1}\right) & q_{2}\left(t_{1}\right) & \ldots & q_{K}\left(t_{1}\right)\\ q_{1}\left(t_{2}\right) & q_{2}\left(t_{2}\right) & \ldots & q_{K}\left(t_{2}\right)\\ \vdots & \vdots & \ddots & \vdots \\ q_{1}\left(t_{S}\right) & q_{2}\left(t_{S}\right) & \ldots & q_{K}\left(t_{S}\right) \end{array}\right]$$

**Figure 3:**
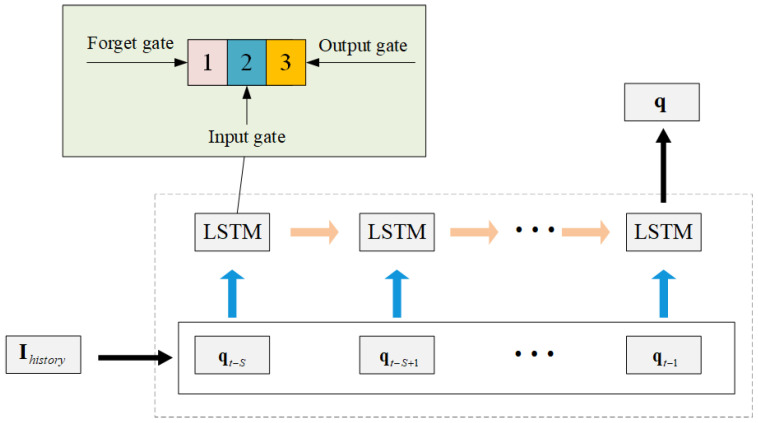
LSTM predicted model.

Based on $I_{\mathrm{history}}$, we utilize LSTM to predict future user positions. The LSTM network is a type of time-recurrent neural network, suitable for processing and predicting sequential data or time series. Take Figure 3 as an example to introduce the composition and functions of the LSTM network unit.

The first part is generally referred to as the forget gate, which is used to determine whether the information from the past time step needs to be forgotten; the second part is called the input gate, through which new information is input into the network; the third part is called the output gate, which is used to pass the information to the next unit. As an excellent perceptron, the LSTM network can fully perceive the relevant features of the time series, such as the temporal correlation between the user’s position information and service changes within a certain period of time, thereby predicting the user’s position at the next moment. The dataset is generated by simulating the random walk trajectories of *K* users within the task area. In each time slot, users move according to a random direction with inertia. The initial direction of users is uniformly distributed within [0,2π], with the turning angle per step constrained to ±π/6. Boundary reflection is implemented to ensure users remain within the task area.

**Figure 4:**
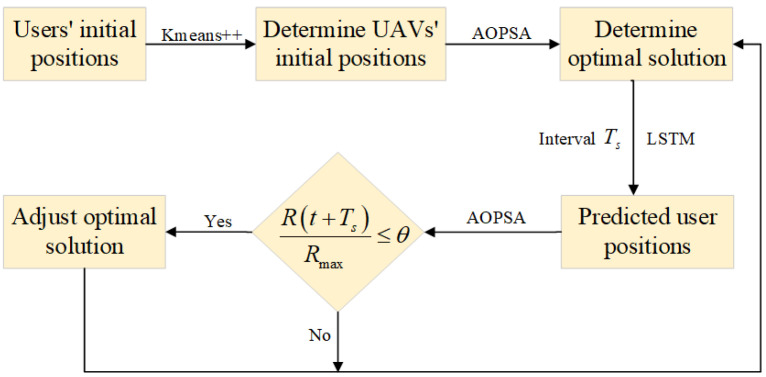
Flowchart of alternating optimization schemes based on user position prediction.

Setting the time interval $T_{s}$ as the cycle for adjusting the optimization scheme, the flowchart of alternating optimization schemes based on user position prediction is shown in Figure 4.

In this figure, $R_{\max}$ represents the objective function value obtained using the optimal optimization scheme for time slot $t+T_{s}$, $R(t+T_{s})$ represents the objective function value obtained, still using the optimal optimization scheme for time slot *t*, and θ is the adjustment threshold, for example 95%.

For the LSTM prediction model, the input size is $I_{\mathrm{history}}$. Let ξ denote the output size and *h* denote the number of units in the hidden layer of the network. According to the connection relationships between neurons, the computational complexity of LSTM can be expressed as $O(4hI_{\mathrm{history}}+4h^{2}+3h+h\xi )$ [26]. We train the LSTM prediction model offline, and then use the trained model for solving the optimization problem to ensure real-time performance.

### 4.2. Determine the Initial Positions of UAVs with Kmeans++

Based on the positions of all users at the initial time slot, we use the Kmeans++ algorithm [27] to determine the initial positions of the UAVs, as shown in Algorithm 1.
**Algorithm 1** Kmeans++ Algorithm**Input:** Users’ initial positions
**Output:** UAVs’ initial positions
1:Randomly select a user as the first center $c_{1}$;2:Calculate the shortest distance $d_{k}$ from ${\mathrm{UE}}_{k}$ to the selected center set C;3:Select a new user as the next center with probability $d_{k}^{2}/\sum_{k=1}^{K}d_{k}^{2}$;4:**Repeat** steps **2–3** until $C=\{c_{1},c_{2},\ldots ,c_{M}\}$;5:Assign each user to the nearest center, forming *M* clusters;6:Calculate the average position of users in each cluster as the new center;7:**Repeat** steps **5–6** until the cluster centers are stable.


### 4.3. User Association Optimization

For given q and p, the user association optimization subproblem can be formulated as

(15)P1:maxαRsumcom(15a)s.t.∑m=1Mαmk(t)≤1,∀k,t(15b)αmk(t)∈{0,1},∀m,k,t(15c)γmkrad(t)≥γthrad,∀m,k,t

The traditional auction algorithm is difficult to adapt to many-to-many association requirements. Therefore, we propose an IAA based on the dynamic pricing mechanism to solve P1, maximizing user rates while ensuring sensing service quality. The process of solving P1 with IAA is described as follows:

We define the price of ${\mathrm{UAV}}_{m}$ as $\psi_{m}^{\mathrm{price}}$, initialize the prices of all UAVs as ${\left[\psi_{1}^{\mathrm{price}},\psi_{2}^{\mathrm{price}},\ldots ,\psi_{M}^{\mathrm{price}}\right]}^{T}\leftarrow 0$, and the set of unassigned users U←{1,2,…,K}.

For each unassigned ${\mathrm{UE}}_{k}\in U$:

- Select the set of valid UAVs that satisfy the sensing constraint
  (16) $$M_{k}=\left\{m|\gamma_{mk}^{\mathrm{rad}}\left(t\right)\geq \gamma_{th}^{\mathrm{rad}}\right\}$$
- Calculate the net benefit for ${\mathrm{UAV}}_{m}\in M_{k}$ to ${\mathrm{UE}}_{k}$
  (17) $$b_{mk}=R_{mk}^{\mathrm{com}}\left(t\right)-\psi_{m}^{\mathrm{price}}$$
- Select the UAV with the maximum net benefit
  (18) $$m^{*}=\arg\max_{m\in M_{k}}b_{mk}$$
- Update the price of $m^{*}$, with the update mechanism as follows
  (19) $$b_{mk}^{\left(2\right)}=\max_{m\in M_{k},m\neq m^{*}}b_{mk}$$
  (20) $$\Delta p_{m^{*}}=\left(b_{m^{*}k}-b_{mk}^{\left(2\right)}\right)+\varepsilon_{\mathrm{basic}}\cdot \left(1+\frac{b_{m^{*}k}-b_{mk}^{\left(2\right)}}{b_{m^{*}k}+\varepsilon_{0}}\right)$$
  where $b_{mk}^{\left(2\right)}$ is the suboptimal net benefit, $\Delta p_{m^{*}}$ is the price increment, $\varepsilon_{\mathrm{basic}}$ is the basic step size, and $\varepsilon_{0}$ prevents division by zero.

The detailed process of solving P1 with IAA is shown in Algorithm 2. The computational complexity of IAA is mainly related to the number of UAVs and users. Therefore, the total computational complexity of IAA is O(KM). This algorithm coordinates resource competition among multiple UAVs by introducing dynamic price adjustment increment $\Delta p_{m^{*}}$, providing an effective solution paradigm for joint optimization problems in dynamic environments. Its distributed characteristics and low computational complexity make it particularly suitable for real-time resource scheduling scenarios in large-scale multi-UAV networks.
**Algorithm 2** Improved Auction Algorithm (IAA)**Input:** $\gamma_{mk}^{\mathrm{rad}}\left(t\right)$, $R_{mk}^{\mathrm{com}}\left(t\right)$, $\gamma_{th}^{\mathrm{rad}}$, $\varepsilon_{\mathrm{basic}}$, q, p **Output:** $\alpha_{\mathrm{best}}$ **Initialize:** U←{1,2,…,K}, ${\left[\psi_{1}^{\mathrm{price}},\psi_{2}^{\mathrm{price}},\ldots ,\psi_{M}^{\mathrm{price}}\right]}^{T}\leftarrow 0$1:**while** U≠⌀ **do**2:   Randomly select ${\mathrm{UE}}_{k}\in U$, filter $M_{k}$ based on Equation (16);3:   **if** $M_{k}=⌀$ **then**4:     Update U=U∖{k};5:   **else**6:     Select UAV $m^{*}$ based on Equations (17) and (18);7:     Update $\alpha_{mk}=1,U=U\setminus \left\{k\right\}$;8:     Update the price of UAV $m^{*}$ based on Equations (19) and (20);9:   **end if**10:**end while**11:**return** $\alpha_{\mathrm{best}}$.

### 4.4. UAV Trajectory Optimization

For given α and p, the UAV trajectory optimization subproblem can be formulated as

(21)P2:maxqRsumcom(21a)s.t.γmkrad(t)≥γthrad,∀m,k,t(21b)xmin≤xm(t)≤xmax,∀m,t(21c)ymin≤ym(t)≤ymax,∀m,t(21d)∥qm(t+1)−qm(t)∥≤vmaxδt,∀t(21e)∥qm(t)−qn(t)∥≥Dmin,∀t,m≠n(21f)pm(t)·δt+κ·∥qm(t+1)−qm(t)∥≤Emax/T,∀m∈M,t∈T

We use the DOA [28] to solve P2. DOA is a novel meta-heuristic algorithm inspired by human dream behavior, which simulates the processes of memory retention, forgetting, and self-organization in dreams. By integrating memory, forgetting, and supplementary strategies, it effectively balances global exploration and local exploitation. The memory strategy serves as the foundation of the algorithm, resetting individuals to the previous optimal values within the group or population. This is followed by the forgetting supplement, which facilitates a gradual shift from exploration to exploitation. In the exploration stage, global optimization is enhanced through grouping, while in the exploitation stage, the entire population collaborates to improve local optimization. Reference [28] demonstrates that compared to algorithms such as particle swarm optimization (PSO) and successive convex approximation (SCA), DOA performs superiorly in terms of convergence, stability, and overall performance. The process of solving P2 with DOA is described as follows.

#### 4.4.1. Optimization Algorithm Assumptions

Based on the characteristics of human dreams and knowledge of optimization algorithms, we summarize the following four hypotheses:

The quality of dreams can be evaluated using fitness values.

The initiation of dreams is closely related to the foundation of existing memories.

Humans partially forget existing memories and supplement the forgotten parts with logically self-organized information.

Memory ability varies among individuals or groups and exhibits a certain degree of randomness.

These hypotheses help us better understand the principles of the algorithm. The workflow, exploration phase, and various strategies in the development phase of the algorithm are concrete embodiments of these four hypotheses.

#### 4.4.2. Initialization

Based on the optimal UAV positions obtained from the last alternating optimization $q^{\mathrm{last}}=\left[x_{1}^{\mathrm{last}}y_{1}^{\mathrm{last}},x_{2}^{\mathrm{last}}y_{2}^{\mathrm{last}},\ldots ,x_{M}^{\mathrm{last}}y_{M}^{\mathrm{last}}\right]$, initialize the population Q and ensure that each individual in Q satisfies constraints (21a)–(21f). Population Q can be expressed as

(22) $$Q=\left[\begin{array}{c} q_{1}\\ q_{2}\\ \vdots \\ q_{N_{1}} \end{array}\right]=\left[\begin{array}{ccccccc} x_{1,1} & y_{1,1} & x_{2,1} & y_{2,1} & \ldots & x_{M,1} & y_{M,1}\\ x_{1,2} & y_{1,2} & x_{2,2} & y_{2,2} & \ldots & x_{M,2} & y_{M,2}\\ \vdots & \vdots & \vdots & \vdots & \ddots & \vdots & \vdots \\ x_{1,N_{1}} & y_{1,N_{1}} & x_{2,N_{1}} & y_{2,N_{1}} & \ldots & x_{M,N_{1}} & y_{M,N_{1}} \end{array}\right]$$

where $N_{1}$ is the number of individuals and $q_{i}$ represents the *i*th individual in Q. Each individual represents the horizontal coordinates of all UAVs, i.e., $\left(x_{m,i},y_{m,i}\right)$ represents the horizontal coordinates of the *m*th UAV in the *i*th individual. The initialization population is formulated as follows

(23) $$x_{m,i}=x_{m}^{\mathrm{last}}+r\times \left(x_{\max}-x_{\min}\right)$$

(24) $$y_{m,i}=y_{m}^{\mathrm{last}}+r\times \left(y_{\max}-y_{\min}\right)$$

where *r* is a random number between 0 and 1.

#### 4.4.3. Fitness Function

Transforming constraints (21a)–(21f) into penalty function terms, the fitness function can be expressed as

(25) $$F_{\mathrm{fitness}}\left(q_{i}\right)=R_{\mathrm{sum}}^{\mathrm{com}}-\varpi_{1}\sum_{v\in V_{q}}\Phi_{v}$$

where $\Phi_{v}$ is the constraint violation indicator function, $\varpi_{1}$ is the penalty coefficient, and $V_{q}$ is the set of constraints (21a)–(21f).

#### 4.4.4. Exploration Phase $\left(0<u\leq u_{d}\right)$

We divide the population into five groups based on the the difference in memory ability, the number of forgetting dimension $f_{F}$ for each group can be expressed as

(26) $$f_{F}=\vartheta_{\mathrm{randi}}\left(\left\lceil \frac{D_{\dim}}{8F}\right\rceil ,\max\left\{2,\left\lceil \frac{D_{\dim}}{3F}\right\rceil \right\}\right)$$

where F=1,2,3,4,5 represents the group number, $\vartheta_{\mathrm{randi}}\left(a,b\right)$ represents a random integer selected from *a* to *b*, and $D_{\dim}=2M$ represents the number of dimensions.

*Memory Strategy:* Each iteration is regarded as a dream behavior. Before each iteration, individuals in each group reset their positions to the best individual from the previous iteration within the group. The update formula can be expressed as

(27) $$q_{i}^{u+1}=q_{{\mathrm{best}}_{F}}^{u}$$

where $q_{i}^{u+1}$ represents the *i*th individual at iteration u+1, and $q_{{\mathrm{best}}_{F}}^{u}$ represents the best individual in group *F* at iteration *u*.

*Forgetting and supplementary strategy:* Individuals in each group randomly forget information in certain dimensions while dreaming. The forgetting and supplementary strategy follows the memory strategy, allowing individuals to forget and self-supplement position information in the forgotten dimensions. The update formula can be expressed as

(28) $$\begin{array}{cc} x_{m,i}^{u+1} & =x_{m,{\mathrm{best}}_{F}}^{u}+\frac{1}{2}\left(x_{m}^{\mathrm{last}}+r\times \left(x_{\max}-x_{\min}\right)\right) \end{array}\;\begin{array}{cc} \; & \;\times \left(\cos\left(\pi \times \frac{u+u_{\max}-u_{d}}{u_{\max}}\right)+1\right) \end{array}$$

(29) $$\begin{array}{cc} y_{m,i}^{u+1} & =y_{m,{\mathrm{best}}_{F}}^{u}+\frac{1}{2}\left(y_{m}^{\mathrm{last}}+r\times \left(y_{\max}-y_{\min}\right)\right) \end{array}\;\begin{array}{cc} \; & \;\times \left(\cos\left(\pi \times \frac{u+u_{\max}-u_{d}}{u_{\max}}\right)+1\right) \end{array}$$

where $x_{m,i}^{u+1}$ and $y_{m,i}^{u+1}$ represent the x and y coordinates of the *m*th UAV of the *i*th individual at iteration u+1, respectively. $x_{m,{\mathrm{best}}_{F}}^{u}$ and $y_{m,{\mathrm{best}}_{F}}^{u}$ represent the x and y coordinates of the *m*th UAV of the best individual in group *F* at iteration *u*, respectively. $u_{\max}$ is the maximum number of iterations, $u_{d}$ is the maximum number of iterations in the exploration phase, and $u_{d}=0.9u_{\max}$.

#### 4.4.5. Exploitation Phase $\left(u_{d}<u\leq u_{\max}\right)$

*Memory strategy:* This phase does not perform grouping. Before each dream, all individuals reset their positions to the best individual from the previous iteration in the entire population. The update formula can be expressed as

(30) $$q_{i}^{u+1}=q_{\mathrm{best}}^{u}$$

where $q_{\mathrm{best}}^{u}$ represents the best individual in the entire population at iteration *u*. The number of forgetting dimensions for all individuals in the population is the same, expressed as

(31) $$f=\vartheta_{\mathrm{randi}}\left(2,\max\left\{2,\left\lceil \frac{D_{\dim}}{3}\right\rceil \right\}\right)$$

*Forgetting and supplementary strategy:* The position update formula can be expressed as

(32) $$\begin{array}{cc} x_{m,i}^{u+1} & =x_{m,\mathrm{best}}^{u}+\frac{1}{2}\left(x_{m}^{\mathrm{last}}+r\times \left(x_{\max}-x_{\min}\right)\right) \end{array}\;\begin{array}{cc} \; & \times \left(\cos\left(\pi \times \frac{u}{u_{\max}}\right)+1\right) \end{array}$$

(33) $$\begin{array}{cc} y_{m,i}^{u+1} & =y_{m,\mathrm{best}}^{u}+\frac{1}{2}\left(y_{m}^{\mathrm{last}}+r\times \left(y_{\max}-y_{\min}\right)\right) \end{array}\;\begin{array}{cc} \; & \;\times \left(\cos\left(\pi \times \frac{u}{u_{\max}}\right)+1\right) \end{array}$$

where $x_{m,\mathrm{best}}^{u}$ and $y_{m,\mathrm{best}}^{u}$ represent the x and y coordinates of the *m*th UAV of the best individual in the entire population at iteration *u*, respectively.

The detailed process of solving P2 with DOA is shown in Algorithm 3. The computational complexity of DOA is mainly concentrated in the exploration phase and exploitation phase. The computational complexity of the exploration phase can be expressed as $O(u_{d}\times N_{1}\times D_{\dim})$, and the computational complexity of the exploitation phase can be expressed as $O(\left(u_{\max}-u_{d}\right)\times N_{1}\times D_{\dim})$. Therefore, the total computational complexity of DOA is $O(u_{\max}\times N_{1}\times D_{\dim})$.
**Algorithm 3** Dream Optimization Algorithm (DOA)**Input:** 
$\gamma_{mk}^{\mathrm{rad}}\left(t\right)$, $R_{mk}^{\mathrm{com}}\left(t\right)$, $\gamma_{th}^{\mathrm{rad}}$, $N_{1}$, $u_{\max}$, α, p
**Output:** 
$q_{\mathrm{best}}$, $F_{\mathrm{fitness}}\left(q_{\mathrm{best}}\right)$
**Initialize:** 
u←1, Generate initial population Q based on Equations (22)–(24)
1:**while** $0<u\leq u_{d}$ **do**2:   **for**  F=1:5 **do**3:      Update $q_{{\mathrm{best}}_{F}}$ and $F_{\mathrm{fitness}}\left(q_{{\mathrm{best}}_{F}}\right)$;4:      Update $f_{F}$ based on Equation (26);5:      Update $q_{i}^{u+1}$ based on Equation (27);6:      **for** each individual i∈F **do**7:         Randomly select $f_{F}$ dimensions to forget;8:         **for** each forgotten dimension *m* **do**9:           Update $x_{m,i}^{u+1}$ and $y_{m,i}^{u+1}$ based on Equations (28) and (29);10:         Check and repair constraints (21a)–(21f) for $x_{m,i}^{u+1}$ and $y_{m,i}^{u+1}$;11:        **end for**12:     **end for**13:   **end for**14:   Update $q_{\mathrm{best}}$ and $F_{\mathrm{fitness}}\left(q_{\mathrm{best}}\right)$ based on Equation (25);15:   Update u=u+1;16:**end while**17:**while** $u_{d}<u\leq u_{\max}$ **do**18:   Update $q_{i}^{u+1}$ based on Equation (30);19:   Update *f* based on Equation (31);20:   **for** each individual *i* **do**21:     Randomly select *f* dimensions to forget;22:     **for** each forgotten dimension *m* **do**23:        Update $x_{m,i}^{u+1}$ and $y_{m,i}^{u+1}$ based on Equations (32) and (33);24:        Check and repair constraints (21a)–(21f) for $x_{m,i}^{u+1}$ and $y_{m,i}^{u+1}$;25:     **end for**26:   **end for**27:   Update $q_{\mathrm{best}}$ and $F_{\mathrm{fitness}}\left(q_{\mathrm{best}}\right)$ based on Equation (25);28:   Update u=u+1;29:**end while**30:**return** $q_{\mathrm{best}}$ and $F_{\mathrm{fitness}}\left(q_{\mathrm{best}}\right)$.


### 4.5. UAV Transmit Power Optimization

For given α and q, the UAV transmit power optimization subproblem can be expressed as

(34)P3:maxpRsumcom(34a)s.t.γmkrad(t)≥γthrad,∀m,k,t(34b)0≤pm(t)≤pmax,∀m,t(34c)pm(t)·δt+κ·∥qm(t+1)−qm(t)∥≤Emax/T,∀m∈M,t∈T

We use RRTOA [29] to solve P3. RRTOA is a novel meta-heuristic algorithm inspired by the search mechanism of rapidly-exploring random trees in robot path planning. The rapidly-exploring random trees algorithm effectively explores the search space by incrementally expanding a tree structure, demonstrating strong adaptability to complex constraints and high-dimensional planning requirements. However, traditional rapidly-exploring random trees algorithms suffer from issues such as slow convergence rates and undesirable path quality. Particularly in regions near the goal, the tree expansion becomes sparse, leading to reduced computational efficiency. To address these limitations, RRTOA has been effectively improved. RRTOA utilizes the adaptive step size wandering strategy, absolute difference-based adaptive step size strategy, and boundary-based adaptive step size strategy, which can effectively search the solution space while guiding the population to find high-quality solutions. Reference [29] shows that compared to similar meta-heuristic algorithms, RRTOA achieves competitive results across various problems. The process of solving P3 with RRTOA is described as follows.

#### 4.5.1. Initialization

Based on the optimal UAV power allocation scheme from the last alternating optimization $p^{\mathrm{last}}=\left[p_{1}^{\mathrm{last}},p_{2}^{\mathrm{last}},\ldots ,p_{M}^{\mathrm{last}}\right]$, initialize population P and ensure individuals in the population satisfy constraints (34a)–(34c). Population P can be expressed as

(35) $$P=\left[\begin{array}{c} p_{1}\\ p_{2}\\ \vdots \\ p_{N_{2}} \end{array}\right]=\left[\begin{array}{cccc} p_{1,1} & p_{2,1} & \ldots & p_{M,1}\\ p_{1,2} & p_{2,2} & \ldots & p_{M,2}\\ \vdots & \vdots & \ddots & \vdots \\ p_{1,N_{2}} & p_{2,N_{2}} & \ldots & p_{M,N_{2}} \end{array}\right]$$

where $N_{2}$ is the number of individuals, each individual $p_{j}$ represents a UAV power allocation scheme. The initialization formula for the population is as follows

(36) $$p_{m,j}=p_{m}^{\mathrm{last}}+r_{1}\times \left(p_{\max}-p_{\min}\right)$$

where $p_{m,j}$ represents the power of the *m*th UAV in the *j*th individual, and $r_{1}$ is a random number between 0 and 1.

#### 4.5.2. Fitness Function

Transforming constraints (34a)–(34c) into penalty function terms, the fitness function is expressed as

(37) $$F_{\mathrm{fitness}}\left(p_{j}\right)=R_{\mathrm{sum}}^{\mathrm{com}}-\varpi_{2}\sum_{v\in V_{p}}\Phi_{v}$$

where $\Phi_{v}$ is the constraint violation indicator function, $\varpi_{2}$ is the penalty coefficient, and $V_{p}$ is the set of constraints (34a)–(34c).

#### 4.5.3. Adaptive Step Size Wandering Strategy

To address the issue of low exploration efficiency caused by a fixed step size, RRTOA models the random sampling mechanism as an adaptive step size wandering strategy to enhance the algorithm’s search capability. The adaptive step size wandering strategy greatly improves the effectiveness of the global search by combining global random initialization methods, effectively preventing the algorithm from falling into local optima. We define monotonic functions $A_{1}$ and $A_{2}$ that vary with the iteration number $u^{\prime }$ to control the dynamic adjustment of the step size, which can be expressed as

(38) $$A_{1}=\frac{\ln(u_{\max}^{\prime }-u^{\prime })}{\ln(u_{\max}^{\prime })},\;A_{2}=\sqrt[{3}]{u^{\prime }/u_{\max}^{\prime }}$$

where $u_{\max}^{\prime }$ is the maximum number of iterations, and $u^{\prime }$ is the current iteration number.

When $r_{1}<A_{1}$, the update formula is as follows

(39) $$p_{m,j}^{u^{\prime }+1}=p_{m,j}^{u^{\prime }}+R_{1}$$

(40) $$R_{1}=\left(r_{1}-\frac{A_{1}}{2}\right)\times A_{1}\times \frac{\left(p_{\max}-p_{\min}\right)}{\hat{\lambda }}$$

where $p_{m,j}^{u^{\prime }+1}$ represents the power of the *m*th UAV in the *j*th individual at iteration $u^{\prime }+1$, $p_{m,j}^{u^{\prime }}$ represents the power of the *m*th UAV in the *j*th individual at iteration $u^{\prime }$, $R_{1}$ is the adaptive step size of this strategy, and $\hat{\lambda }=10$ is the step size penalty factor.

#### 4.5.4. Absolute Difference-Based Adaptive Step Size Strategy

RRTOA introduces an absolute difference-based adaptive step size strategy. This strategy dynamically adjusts the step size by calculating the absolute difference between the current particle position and the current best position, achieving a dynamic balance between large-scale exploration and local fine-tuning of the search space. Consequently, it enhances the algorithm’s global optimization capability and efficiency.

When $r_{2}<\left(A_{2}/10\right)$, the update formula is as follows

(41) $$p_{m,j}^{u^{\prime }+1}=p_{m,\mathrm{best}}^{u^{\prime }}+R_{2}$$

(42) $$R_{2}=a_{1}\left|p_{m,\mathrm{best}}^{u^{\prime }}-p_{m,j}^{u^{\prime }}\right|$$

where $p_{m,\mathrm{best}}^{u^{\prime }}$ represents the power of the *m*th UAV in the best individual at iteration $u^{\prime }$, $R_{2}$ is the adaptive step size of this strategy, $r_{2}$ is a random number between 0 and 1, and $a_{1}$ is the adaptive step size adjustment coefficient of this strategy, which can be expressed as

(43) $$a_{1}=5\times \left(r_{2}-A_{2}/20\right)\times \cos\left(2\pi r_{2}\right)\times e^{b}$$

where $e^{b}$ is the random disturbance factor, with $b=e^{\cos(\pi -\pi /u^{\prime })}$.

#### 4.5.5. Boundary-Based Adaptive Step Size Strategy

To achieve more precise optimization, a boundary-based adaptive step size strategy is introduced. The core of this strategy involves exploring with very small random steps, which enables RRTOA agents to conduct meticulous searches in the surrounding area. This not only ensures localized searching around the current best solution but also prevents convergence to local minima.

When $r_{3}<\left(A_{2}/50\right)$, the update formula is as follows

(44) $$p_{m,j}^{u^{\prime }+1}=p_{m,\mathrm{best}}^{u^{\prime }}+R_{3}$$

(45) $$R_{3}=\left(p_{\max}-p_{\min}\right)\times \cos\left(10\pi u^{\prime }/u_{\max}^{\prime }\right)\times a_{2}$$

where $R_{3}$ is the adaptive step size of this strategy, $r_{3}$ is a random number between 0 and 1, and $a_{2}$ is the adaptive step size adjustment coefficient of this strategy, which can be expressed as

(46) $$a_{2}=r_{3}\times \left(r_{3}-A_{2}/100\right)\times A_{1}\times \left(1-u^{\prime }/u_{\max}^{\prime }\right)$$

The detailed process of solving P3 with RRTOA is shown in Algorithm 4. The computational complexity of RRTOA is mainly affected by population size, number of iterations, and problem dimension. Therefore, the total computational complexity of RRTOA is $O(u_{\max}^{\prime }\times N_{2}\times M)$.
**Algorithm 4** RRT-Based Optimizer Algorithm (RRTOA)**Input:** 
$\gamma_{mk}^{\mathrm{rad}}\left(t\right)$, $R_{mk}^{\mathrm{com}}\left(t\right)$, $\gamma_{th}^{\mathrm{rad}}$, $N_{2}$, $u_{\max}^{\prime }$, α, q
**Output:** 
$p_{\mathrm{best}}$, $F_{\mathrm{fitness}}\left(p_{\mathrm{best}}\right)$
**Initialize:** 
$u^{\prime }\leftarrow 1$, generate initial population P based on Equations (35) and (36)
1: 
**while** $u^{\prime }\leq u_{\max}^{\prime }$ **do**2: 
   **if**  $r_{1}<A_{1}$ **then**3: 
     Update $p_{m,j}^{u^{\prime }+1}$ based on Equations (39) and (40);4: 
   **end if**5: 
   **if**  $r_{2}<\left(A_{2}/10\right)$ **then**6: 
     Update $p_{m,j}^{u^{\prime }+1}$ based on Equations (41)–(43);7: 
   **end if**8: 
   **if**  $r_{3}<\left(A_{2}/50\right)$ **then**9: 
     Update $p_{m,j}^{u^{\prime }+1}$ based on Equations (44)–(46);10:   **end if**11:   Update $p_{\mathrm{best}}$ and $F_{\mathrm{fitness}}\left(p_{\mathrm{best}}\right)$ based on Equation (37);12:   Update $u^{\prime }=u^{\prime }+1$;13:**end while**14:**return** $p_{\mathrm{best}}$ and $F_{\mathrm{fitness}}\left(p_{\mathrm{best}}\right)$.


### 4.6. Alternating Optimization-Based Predictive Scheduling Algorithm

We define the relevant variables in AOPSA: ${\hat{\mu }}_{\max}$ is the maximum number of iterations, ε is the convergence threshold, and $R_{\mathrm{sum}}^{\mathrm{com}}\left(\hat{\mu }\right)$ represents the objective function value at iteration $\hat{\mu }$. The detailed process of solving P0 with AOPSA is shown in Algorithm 5. The computational complexity of AOPSA is $O\left({\hat{\mu }}_{\max}\times \left(u_{\max}\times N_{1}\times D_{\dim}+u_{\max}^{\prime }\times N_{2}\times M+KM\right)\right)$.
**Algorithm 5** Alternating Optimization-based Predictive Scheduling Algorithm (AOPSA)**Input:** 
*M*, *K*, ${\hat{\mu }}_{\max}$, ε, $\gamma_{mk}^{\mathrm{rad}}\left(t\right)$, $R_{mk}^{\mathrm{com}}\left(t\right)$, $\gamma_{th}^{\mathrm{rad}}$, $N_{1}$, $N_{2}$
**Output:** 
α, q, p, $R_{\mathrm{sum}}^{\mathrm{com}}$
**Initialize:** UAV initial positions, $p\leftarrow p_{\max}$, α←0, $R_{\mathrm{sum}}^{\mathrm{com}}\leftarrow 0$
1: **for** $\hat{\mu }=1$: ${\hat{\mu }}_{\max}$ **do**2:    Optimize user association α using Algorithm 2;3:    Optimize UAV trajectory q using Algorithm 3;4:    Optimize UAV transmit power p using Algorithm 4;5:    Calculate $R_{\mathrm{sum}}^{\mathrm{com}}\left(\hat{\mu }\right)$;6:    **if**  $|R_{\mathrm{sum}}^{\mathrm{com}}\left(\hat{\mu }\right)-R_{\mathrm{sum}}^{\mathrm{com}}|<\varepsilon$ **then**7:      **break**;8:    **else**9:      Update $R_{\mathrm{sum}}^{\mathrm{com}}\leftarrow R_{\mathrm{sum}}^{\mathrm{com}}\left(\hat{\mu }\right)$;10:   **end if**11:**end for**12:**return** α, q, p, $R_{\mathrm{sum}}^{\mathrm{com}}$.


## 5. Simulation Results

This section evaluates the performance of AOPSA through simulation. Regarding the frequency selection, we adopt the millimeter-wave frequency band (28 GHz). This frequency band has abundant spectrum resources and can meet the system’s requirements for high-speed data transmission and high-precision sensing. The initial positions of ground users are randomly distributed within a 2D area of 800 m × 800 m. The main simulation parameters are shown in Table 1, and the settings of the values mainly refer to [16,25].

To demonstrate the effectiveness of AOPSA, we compare AOPSA with three other schemes as well as the joint optimization scheme in [30]. The details are as follows:

**Scheme 1**: UAVs are uniformly distributed throughout the area and remain stationary at altitude *H*. Only user association optimization and UAV power optimization are performed.

**Scheme 2**: UAV power is fixed at $p_{\max}$. Only user association optimization and UAV trajectory optimization are performed.

**Scheme 3**: UAVs are uniformly distributed throughout the area and remain stationary at altitude *H*. UAV power is fixed at $p_{\max}$, and only user association optimization is performed.

**Reference [30]**: Adopt spectral clustering, coalition game, and SCA to sequentially solve the three subproblems of UAV position optimization, user association optimization, and transmit power optimization.

We compare and analyze the computational complexity of AOPSA and the reference [30] algorithm as shown in Table 2. The complexity analysis for AOPSA has been presented in Section 4 and will not be reiterated here. The reference [30] algorithm employs coalition game, spectral clustering, and SCA to sequentially solve three subproblems: user association optimization, UAV position optimization, and transmit power optimization. In the user association optimization subproblem, the coalition game requires traversing all possible user–UAV combinations in the worst case, resulting in exponential computational complexity $O(M^{K})$ for solving subproblem P1. The UAV trajectory optimization subproblem involves three components: similarity matrix construction $O(K^{2})$, eigen decomposition $O(K^{3})$, and initial clustering O(KM). For the power optimization subproblem, the computational complexity reaches $O(M^{3.5})$. This analysis reveals that AOPSA significantly reduces the computational complexity and is suited for large scale scenarios.

**Figure 5:**
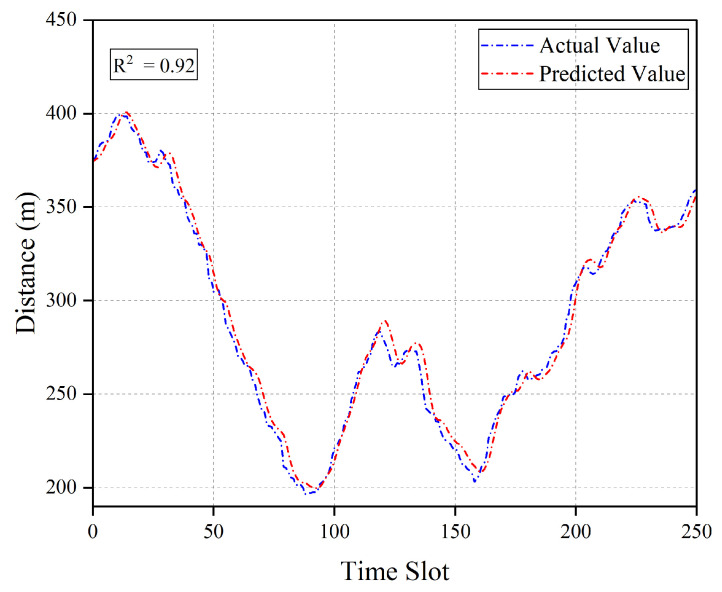
Comparison of LSTM-predicted values and actual values for a single user position.

Figure 5 describes the comparison result of the LSTM-predicted values and actual values for a single user position. To more intuitively present the user position at each time slot, we further convert the user coordinates into the distance between the user and the jammer. As shown in Figure 5, the two curves of predicted values and actual values achieve good fitting (coefficient of determination $R^{2}=0.92$). This indicates that the LSTM model can effectively capture the temporal features of user position changes and achieve relatively accurate predictions.

**Figure 6:**
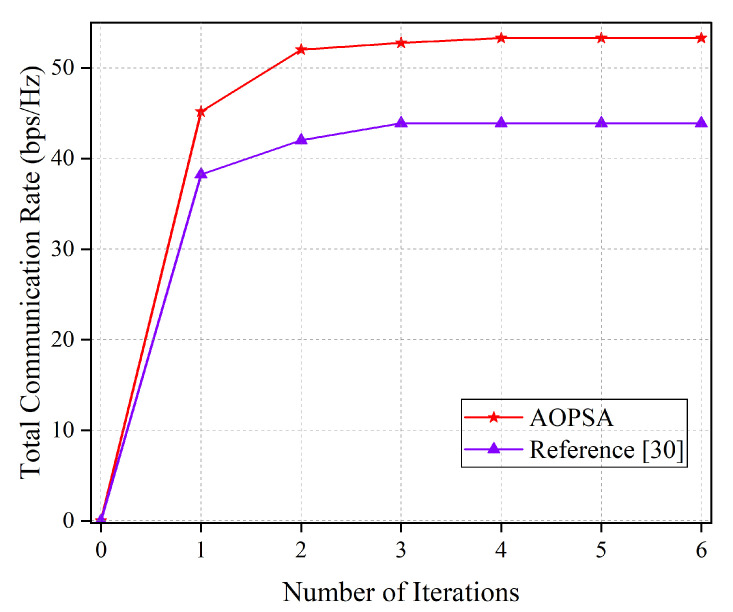
Convergence analysis [30].

Before comparing the performance of different schemes, we validate the convergence behavior of the AOPSA. As illustrated in Figure 6, we compare the total communication rate of AOPSA (per single time slot) with that of the algorithm in reference [30] as a function of iteration numbers. The results demonstrate that AOPSA rapidly increases the communication rate to 53 bps/Hz within the first four iterations and maintains stable performance thereafter. Compared to reference [30], our proposed algorithm achieves approximately 23.2% performance improvement, demonstrating favorable convergence behavior and effectiveness.

**Figure 7:**
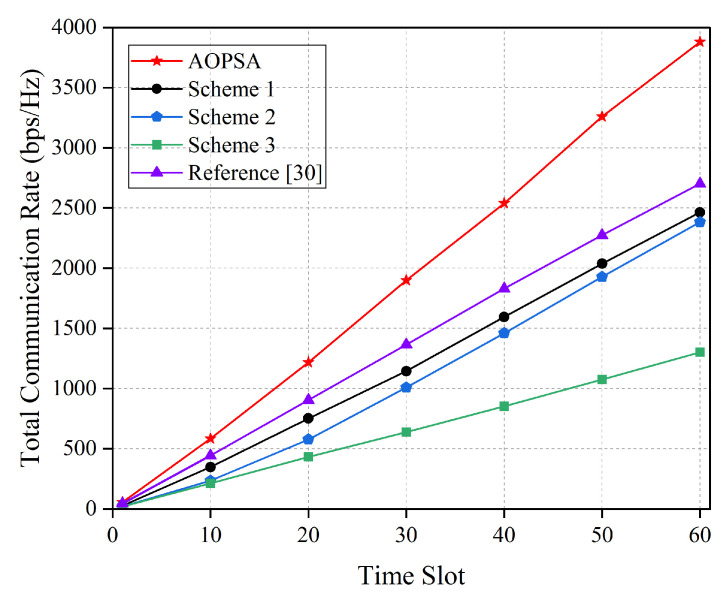
Comparison of total communication rate for five schemes (M=5, K=20) [30].

Figure 7 shows the comparison of total communication rate for five optimization schemes over the entire flight time. It can be observed that as the number of time slots increases, the total communication rates of all schemes show an upward trend. The best performance is achieved by AOPSA, which reaches approximately 3750 bps/Hz at 60th time slot. Reference [30] reaches 2650 bps/Hz, while baseline scheme 3 is only around 1300 bps/Hz. Compared with [30], AOPSA achieves a 41.5% performance improvement, and compared with baseline scheme 3, the performance improvement reaches up to 188%. This result fully demonstrates AOPSA’s effectiveness in solving the joint optimization problem of user association, UAV trajectory optimization, and power allocation.

**Figure 8:**
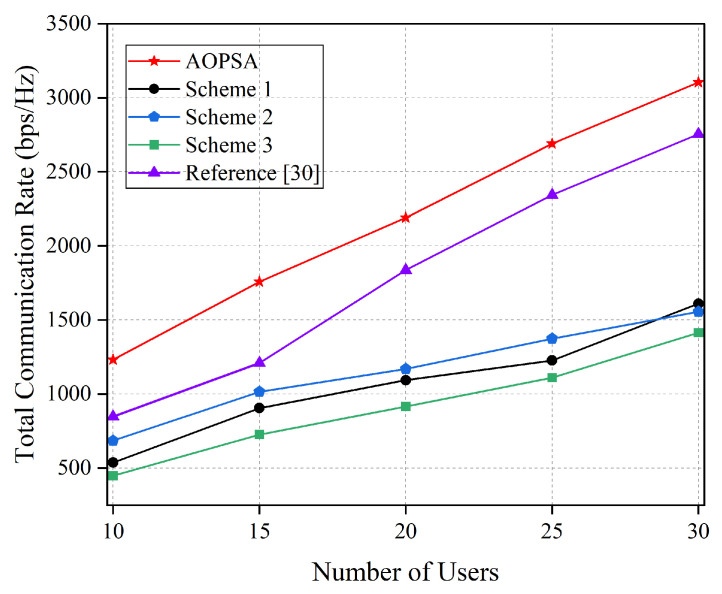
Comparison of total communication rate for five schemes under different numbers of users (M=5) [30].

Figure 8 compares the variation trend of the total communication rate for five optimization schemes under different numbers of users. It can be seen that with the increase in the number of users, the total communication rates of all schemes show an upward trend. AOPSA consistently maintains the best performance, demonstrating higher scalability. This indicates that AOPSA has stronger resource optimization and scheduling capabilities when dealing with multiple scenarios, and can effectively cope with challenges brought by changes in the number of users, providing a reliable performance guarantee for practical deployments.

**Figure 9:**
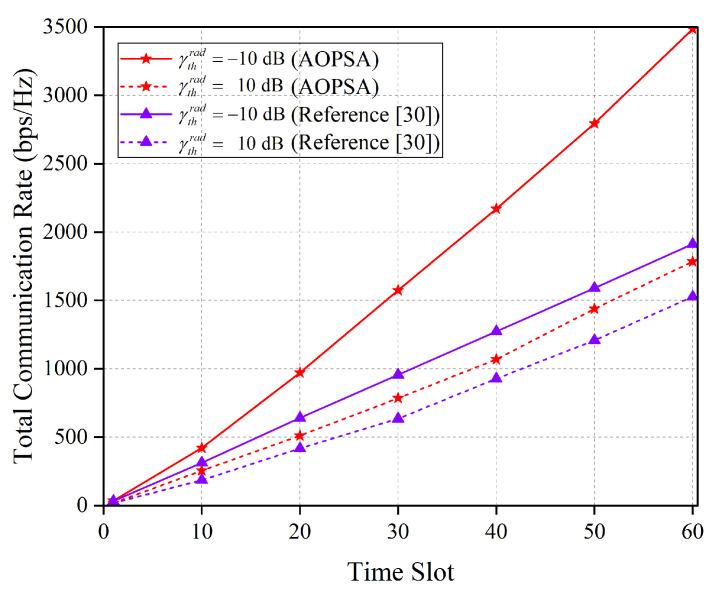
Comparison of the total communication rate between AOPSA and reference [30] under different values of $\gamma_{th}^{\mathrm{rad}}$ (M=5, K=20).

Figure 9 illustrates the performance comparison between the AOPSA and the reference [30] algorithm in terms of the total communication rate over all time slots under different $\gamma_{th}^{rad}$. It can be observed that under the same $\gamma_{th}^{rad}$ constraint, the total communication rate of AOPSA is significantly higher than that of the reference [30] algorithm. As the $\gamma_{th}^{rad}$ value increases, the total communication rates of both algorithms exhibit a decreasing trend. This result indicates that a higher $\gamma_{th}^{rad}$ value requires more resources to meet the sensing performance requirements, thereby leading to a degradation in communication performance. Nevertheless, AOPSA still demonstrates superior performance, achieving an effective balance between communication and sensing.

**Figure 10:**
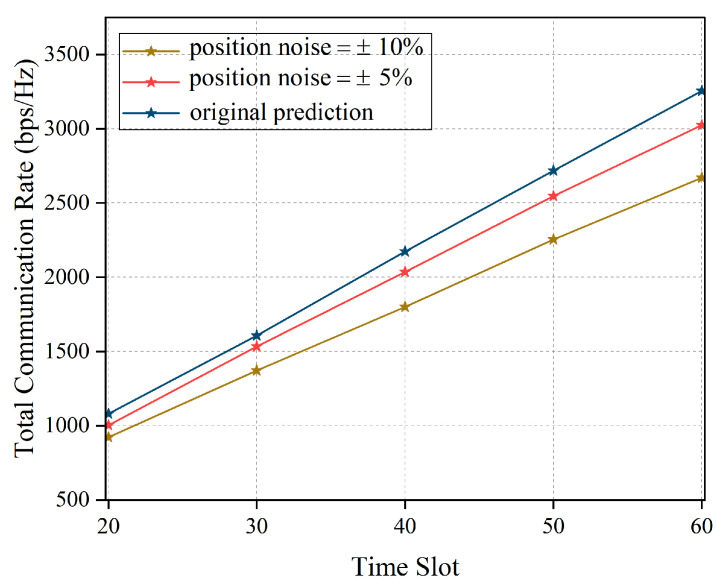
Comparison of total communication rate of AOPSA under different position noise (M=5, K=20).

As shown in Figure 10, we compare the total communication rate of AOPSA under different prediction position noise conditions across time slots. It can be observed that the blue curve represents performance under ideal prediction conditions, with the communication rate increasing from approximately 1100 bps/Hz to 3300 bps/Hz while maintaining the highest transmission efficiency. In practical scenarios with prediction position noise, system performance decreases to varying degrees: the red curve (position noise ±5%) shows communication rates ranging from 1000 bps/Hz to 3000 bps/Hz; the yellow curve (position noise ±10%) exhibits the lowest performance, with rates increasing from 900 bps/Hz to 2600 bps/Hz. This demonstrates that position prediction accuracy significantly impacts system communication performance. As position noise increases, the deviation between system performance and ideal prediction gradually enlarges, further emphasizing the importance of adopting robust prediction algorithms in practical applications.

**Figure 11:**
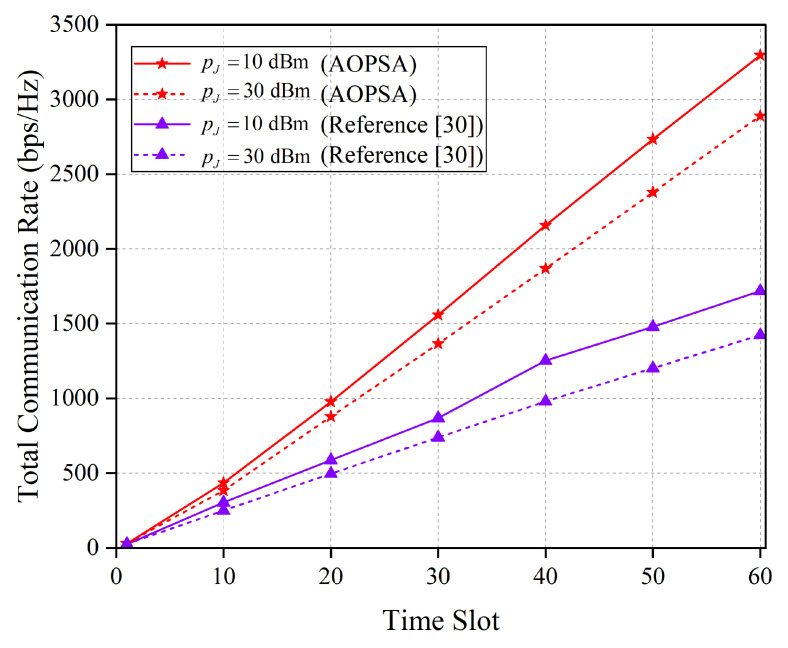
Comparison of the total communication rate between AOPSA and reference [30] under different values of $p_{J}$ (M=5, K=20).

As shown in Figure 11, the total communication rate of the AOPSA and the algorithm in reference [30] is compared across all time slots under different $p_{J}$ values. The results demonstrate that as $p_{J}$ increases, the performance of both algorithms declines. Nevertheless, AOPSA consistently outperforms the reference [30] algorithm. This indicates that the jamming power $p_{J}$ directly affects the SINR of the communication link. Specifically, an increase in $p_{J}$ intensifies the jamming imposed by the jammer on the user, leading to a reduction in the communication SINR and a consequent significant decrease in the communication rate.

**Figure 12:**
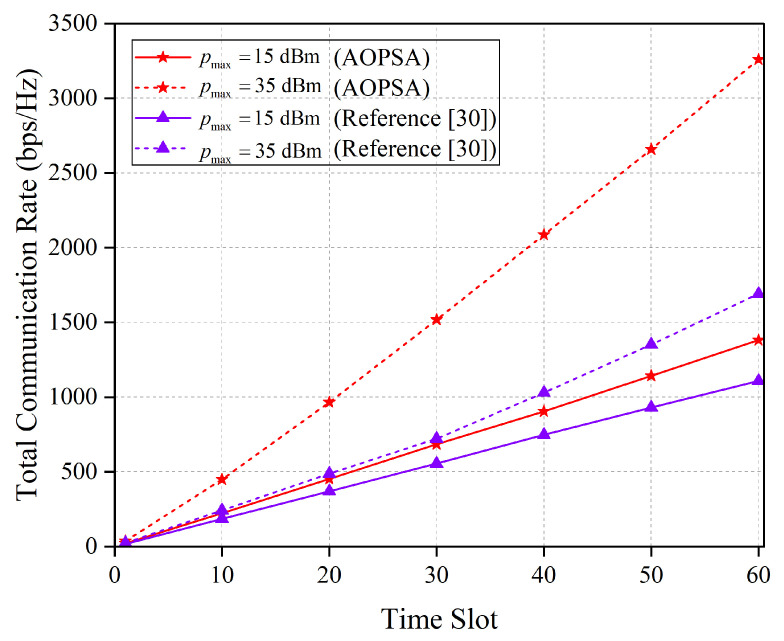
Comparison of the total communication rate between AOPSA and reference [30] under different values of $p_{\max}$ (M=5, K=20).

Figure 12 presents a comparison of the total communication rate between AOPSA and the algorithm in reference [30] across all time slots under different $p_{\max}$ values. As shown in the figure, the performance of both algorithms improves as $p_{\max}$ increases, with AOPSA consistently outperforming the reference [30] algorithm. This result indicates that a higher $p_{\max}$ value expands the feasible region for optimizing the UAV transmit power, allowing the algorithm to more flexibly adjust the power allocation within the extended constraint, thereby achieving significant performance enhancement.

## 6. Conclusions

This paper focuses on the multi-UAV-assisted ISAC system under malicious jamming environments, studying the joint optimization problem of user association, UAV trajectory planning, and transmit power allocation. To address the impact of user mobility and enhance system real-time performance, this paper proposes a user position prediction model based on LSTM to achieve accurate prediction of user positions in future time slots. To reduce the impact of malicious jamming and maximize the total communication rate of users, this paper adopts IAA, DOA, and RRTOA to optimize user association, UAV trajectory, and UAV transmit power, respectively. On this basis, the AOPSA is proposed to achieve joint optimization of the three variables. Simulation results demonstrate that the proposed algorithms can significantly improve the system’s total communication rate under malicious jamming environments, providing reliable technical support for practical deployment.

However, this paper primarily focuses on the analysis and discussion of stationary jammers. In practical scenarios, jammers are often mobile and possess a certain level of intelligence, enabling them to dynamically adjust their jamming methods and positions based on the countermeasures adopted by the communication system. This significantly enhances jamming efficiency and concealment. Such dynamic and intelligent jamming behaviors pose more severe challenges to existing anti-jamming schemes. Therefore, in future work, we will further investigate the underlying mechanisms of dynamic intelligent jamming scenarios and, on this basis, develop corresponding intelligent anti-jamming and collaborative communication strategies to improve the robustness of the system in such dynamic and intelligent jamming environments. 

## Figures and Tables

**Table 1:** Main simulation parameters.

Parameter	Symbol	Value
UAV transmit antenna gain	$G_{s}$	40 dBi
UAV receive antenna gain	$G_{e}$	30 dBi
Jammer transmit antenna gain	$G_{J}$	20 dBi
User receive antenna gain	$G_{r}$	5 dBi
Number of time slots	*T*	60
Length of time slot	$\delta_{t}$	0.5 s
RCS of target	σ	1 $m^{2}$
UAV collision avoidance distance	$D_{\min}$	30 m
UAV maximum speed	$v_{\max}$	30 m/s
UAV flying altitude	*H*	100 m
Noise power	$\sigma_{\mathrm{noise}}^{2}$	−110 dBm
UAV maximum transmit power	$p_{\max}$	35 dBm
Jammer transmit power	$p_{J}$	20 dBm
Minimum SINR for radar sensing	$\gamma_{th}^{\mathrm{rad}}$	−10 dB
Convergence threshold	ε	0.001
Maximum number of iterations	${\hat{\mu }}_{\max}$	15
Allocation weight parameter	*ℓ*	0.5

**Table 2:** Comparison of computational complexity.

	AOPSA	Reference [30]
Subproblem P1	O(KM)	$O(\mu_{1}\times K\times M^{K})$
Subproblem P2	$O\left(u_{\max}\times N_{1}\times D_{\dim}\right)$	$O\left(K^{3}+K^{2}+KM\right)$
Subproblem P3	$O\left(u_{\max}^{\prime }\times N_{2}\times M\right)$	$O\left(\mu_{2}\times M^{3.5}\right)$
Problem P0	$\begin{array}{cc} & O({\hat{\mu }}_{\max}(u_{\max}\times N_{1}\times D_{\dim}+\\ & u_{\max}^{\prime }\times N_{2}\times M+KM)) \end{array}$	$\begin{array}{cc} & O(\eta_{\max}(\mu_{1}\times K\times M^{K}+K^{3}+\\ & \mu_{2}\times M^{3.5})) \end{array}$

## Data Availability

The original contributions presented in this study are included in the article. Further inquiries can be directed to the corresponding author.

## Abbreviations

The following abbreviations are used in this manuscript:

UAV	Unmanned aerial vehicle
ISAC	Integrated sensing and communication
6G	Sixth generation
LoS	Line-of-sight
MI	Mutual information
RIS	Reconfigurable intelligent surface
IoT	Internet of Things
3D	Three-dimensional
AoI	Age of information
CSI	Channel state information
TDM	Time-division multiplexing
RCS	Radar cross section
OFDMA	Orthogonal frequency division multiple access
SINR	Signal-to-interference-plus-noise ratio
AWGN	Additive white Gaussian noise
MINLP	Mixed integer nonlinear programming
LSTM	Long short-term memory
IAA	Improved auction algorithm
PSO	Particle swarm optimization
SCA	Successive convex approximation
DOA	Dream optimization algorithm
RRTOA	Rapidly-exploring random trees-based optimizer algorithm
